# Spitting for Science: Danish High School Students Commit to a Large-Scale Self-Reported Genetic Study

**DOI:** 10.1371/journal.pone.0161822

**Published:** 2016-08-29

**Authors:** Georgios Athanasiadis, Frank G. Jørgensen, Jade Y. Cheng, Peter C. Kjærgaard, Mikkel H. Schierup, Thomas Mailund

**Affiliations:** 1 Bioinformatics Research Centre, Aarhus University, 8000, Aarhus, Denmark; 2 Centre for Biocultural History, Aarhus University, 8000, Aarhus, Denmark; 3 Tørring Gymnasium, 7160, Tørring, Denmark; 4 Department of Culture and Society, Aarhus University, 8000, Aarhus, Denmark; 5 The Natural History Museum of Denmark, University of Copenhagen, 1471, Copenhagen, Denmark; 6 Department of Bioscience, Aarhus University, 8000, Aarhus, Denmark; University of Illinois at Urbana-Champaign, UNITED STATES

## Abstract

Scientific outreach delivers science to the people. But it can also deliver people to the science. In this work, we report our experience from a large-scale public engagement project promoting genomic literacy among Danish high school students with the additional benefit of collecting data for studying the genetic makeup of the Danish population. Not only did we confirm that students have a great interest in their genetic past, but we were also gratified to see that, with the right motivation, adolescents can provide high-quality data for genetic studies.

## Introduction

Genomic literacy among the general public remains a hot topic in the era of precision medicine and translational genomics [1–4], and it encompasses knowledge about the biochemistry and structure of the genome, basic rules of heredity, the polygenic nature of common diseases, the interaction between genes and environment etc. In addition to imparting basic scientific knowledge, genomic literacy covers ethical, legal, and social implications (ELSI) that are essential in order for individuals to make informed decisions about disease [5] or to understand genetic ancestry.

Initiatives promoting genomic literacy can involve any segment of society. In a typical scenario, researchers partner up with public entities with the support of third-party funding agencies [6]. Denmark has a long tradition of supporting such partnerships. Every year in December, the Danish Ministry of Education invites groups involved in scientific outreach and education to apply for funding from the profits of *Danske Spil–*the national lottery in Denmark. The selection of applicants is straightforward, allowing work on the ground to start within a six-month timeframe.

Inspired by the increasing need for genomic literacy, we designed *Where Are You From*? (http://hvorkommerdufra.dk/)–a high school genomic project aiming at (i) demystifying genetic concepts regarding human ancestry; (ii) building bridges between academia and young students interested in pursuing a scientific career; and (iii) collecting high-quality data for genetic analysis. Such data can provide insights into e.g. the genetic affinity of the Danish population with other European populations or historical patterns of admixture and migration, thus improving our knowledge about the genetic history of Denmark [7]; they can also help us build and validate genetic predictors of anthropometric traits like height and body mass index, thus providing a translational framework for improving genetic risk assessment [7]. In brief, the project consisted of collecting DNA and anthropometric data, and included two symposia at the Aarhus University campus, where basic genetic concepts and preliminary scientific results were presented to the students.

## Materials and Methods

### Outreach

**Fig 1:**
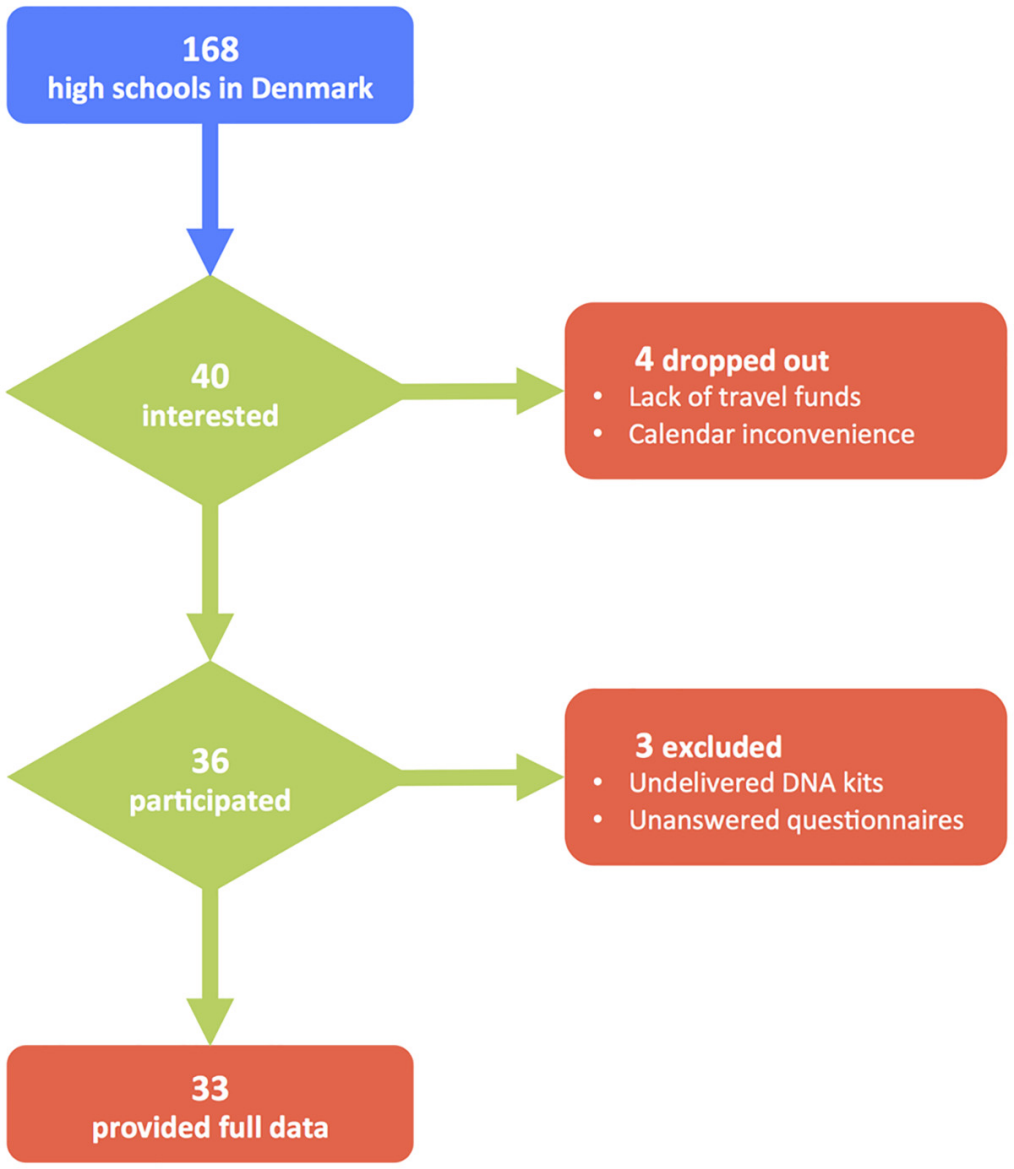
Flowchart of the *Where Are You From*? project. The flowchart displays recruitment, dropout and exclusion rates, as well as tentative reasons for the latter two.

Once funds from *Danske Spil* were secured, we published an official advertisement in a Danish magazine for high school biology teachers, inviting schools from across Denmark to participate in our project [8]. The response was overwhelming: teachers from as many as 40 out of a total of 168 high schools in the entire country expressed initial interest in volunteering for the project (Fig 1). Most of the partners were general high schools (*gymnasium* or *STX* in Danish) with the exception of two technical high schools (*teknisk gymnasium* or *HTX*). Because of the educational structure in Denmark, STX students have a greater than average interest in science and technology. This implies that, to our knowledge, our project was one of the largest gatherings of students with a potential interest in genetics in the history of Danish education.

**Fig 2:**
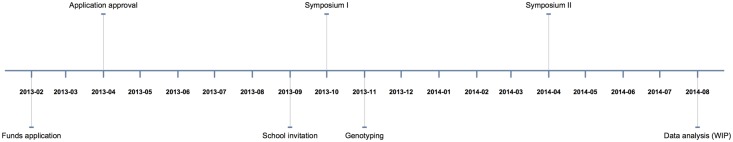
Timeline of the *Where Are You From*? project.

The project included two symposia that took place at the Aarhus University campus–one before and one after data collection (Fig 2)–and covered several topics of genomic analysis applied to humans. In particular, during the first symposium, the students had the chance to learn about basic concepts of human evolution, which included natural selection, the use of mtDNA and Y-chromosome haplotypes to track migration patterns, the use of recombination to study human history and relatedness, the admixture of anatomically modern humans with Neanderthals and Denisovans, but also about genotyping technologies and their potential use in association mapping. During the second symposium, we revisited many of the concepts from the first symposium and we presented preliminary results of the genetic analysis of the students’ DNA.

### Data collection

We asked students to prepare an Oragene•Dx^®^ saliva collection kit from Genotek (Ottawa, Canada) for DNA analysis during science class under the supervision of their teachers and to respond to an online questionnaire about their age; their own, their parents’ and their grandparents’ place of birth; their family’s level of education; and some basic anthropometric traits (S1 Table). We outsourced genotyping to 23andMe (Mountain View, CA, USA) on the grounds of cost-effectiveness and user-friendliness. Initially, students activated their 23andMe profiles, but long before the company returned any genetic results to the users, we changed passwords for all profiles thus preventing access to health-related information outside this project’s scope. We also minimized our own access to health-related information by assigning the entire database handling to a single individual.

### Consent and ethics approval

All students gave their written informed consent to participate in the project, signed either by themselves (>18 years old) or by their parents/guardians (S2 Table). The institutional review board of Aarhus University approved the study. The Regional Research Ethics Committee of Central Jutland informed us orally that no additional medical ethics approval would be required provided that our project entailed no health-related research. Indeed, our questionnaire did not include any health-related questions, and participants had no access to health-related information from their 23andMe profiles (see previous section). A full guideline for medical ethics approval for research in Denmark can be found at http://www.rm.dk/sundhed/faginfo/forskning/de-videnskabsetiske-komiteer/ (in Danish).

## Results

### Participation rates

**Fig 3:**
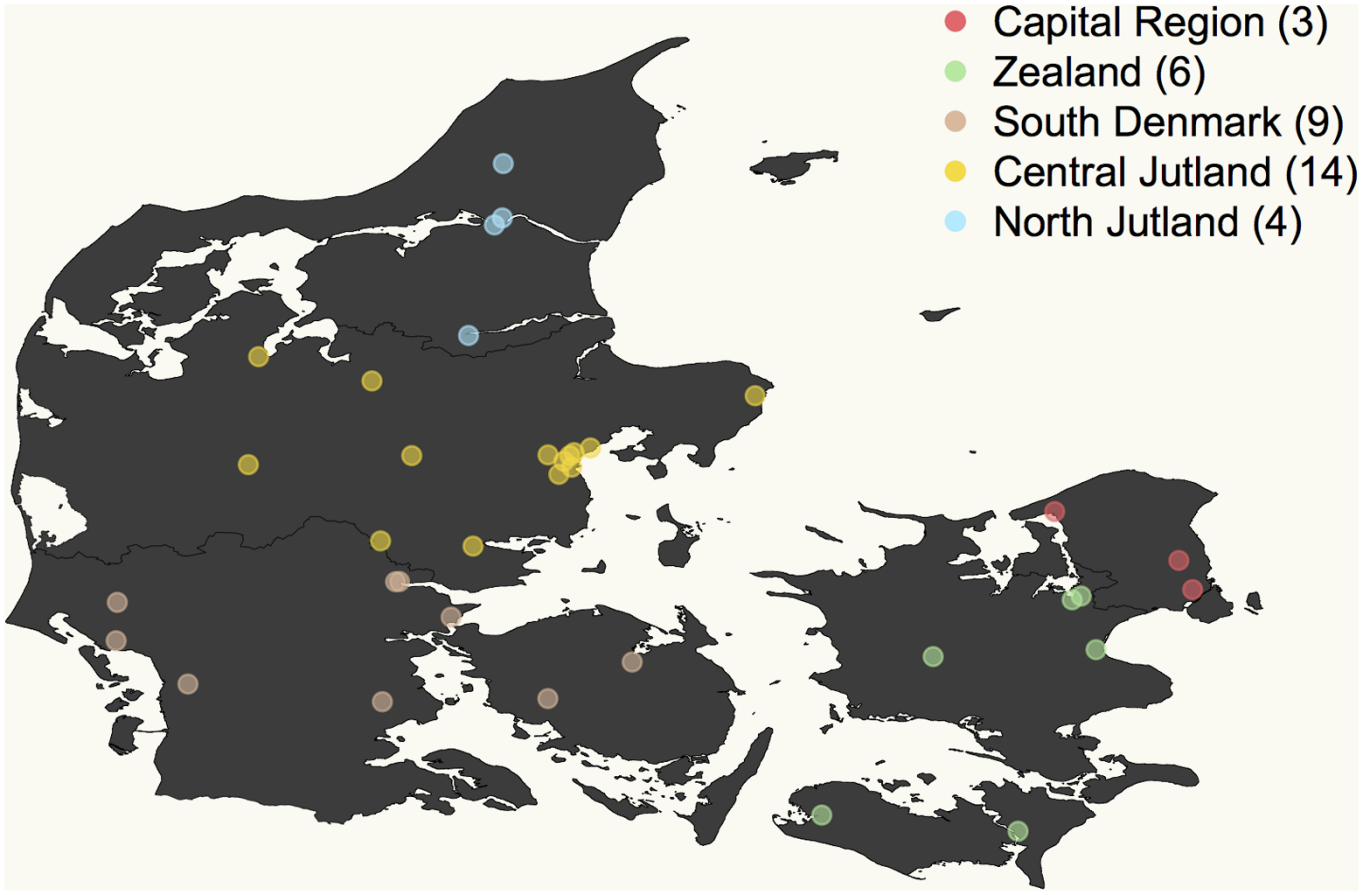
Geographic distribution of the 36 Danish high schools participating in the *Where Are You From*? project. Color code reflects the five administrative divisions of Denmark. Number of schools per division is shown in brackets. Jutland (and Aarhus in particular) high schools were overrepresented in the sampling, due to their proximity to the symposium site in Aarhus University campus.

**Fig 4:**
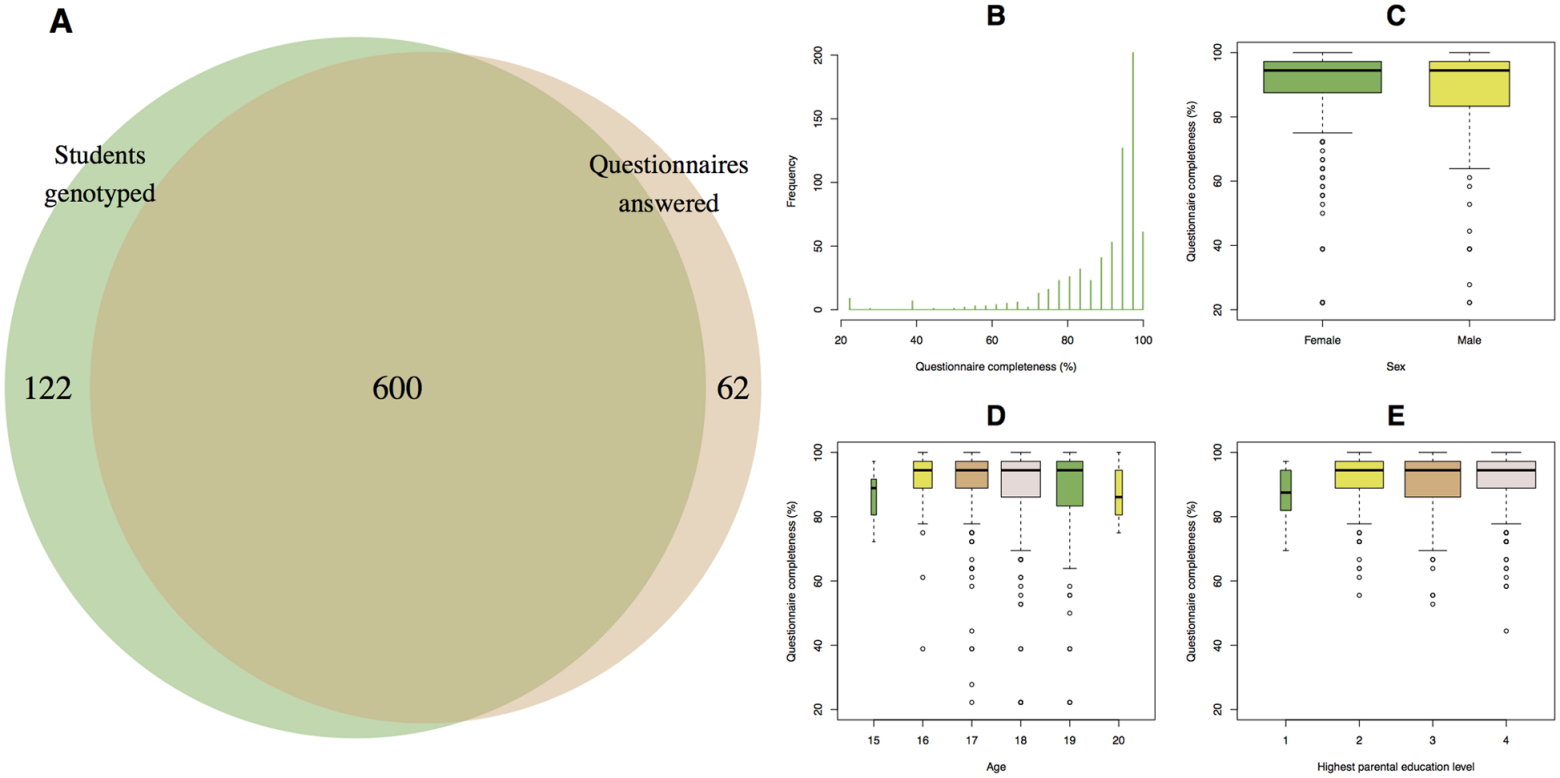
Summary of sample recruitment. (A) Six hundred students from 33 different schools provided both genetic and questionnaire data. (B) Histogram of % questionnaire completeness (N = 662). (C-E) Box plots (median and interquartile range) of % questionnaire completeness by sex, age and highest parental education level (1 = unskilled; 2 = skilled; 3 = short-term higher education; 4 = long-term higher education). Whiskers represent data within 1.5 times the interquartile range and circles represent outliers. Per-individual completeness was defined as the number of fields completed with valid answers over the total number of fields.

Of the 40 schools that initially contacted us, 36 engaged in the activities of the project–i.e. data collection and attendance at the symposia (Table 1). Dropout was primarily due to calendar inconvenience or lack of travel funds. A total of ~1,100 students from the 36 schools volunteered for our project, but because there were not enough funds to cover the genotyping of all of them, we prioritized ~800 while trying to maximize representation of all regions of Denmark (Fig 3). However, regardless of whether they were sampled or not, all students were invited to the outreach activities. After genotyping was completed, we had access to genetic data from 722 kits and information from 662 questionnaires, with 600 students from 33 schools providing both saliva samples and answers to the questionnaire (Fig 4A). To avoid coercion, we did not investigate the reasons for dropping out.

**Table 1:** Name, geographic coordinates and number of questionnaire respondents (N) for each of the 36 schools participating in the *Where Are You From*? project.

High school name	Latitude	Longitude	N respondents
Aalborg Katedralskole	57.05	9.91	18
Aarhus Statsgymnasium	56.16	10.17	13
Brønderslev Gymnasium	57.28	9.94	16
Egaa Gymnasium	56.21	10.27	20
Esbjerg Gymnasium	55.49	8.49	22
Fredericia Gymnasium	55.58	9.75	20
Frederiksbjerg Gymnasium	55.68	12.53	24
Frederiksværk Gymnasium og HF	55.97	12.01	8
Grenaa Gymnasium	56.41	10.89	11
Haderslev Katedralskole	55.26	9.49	29
Herningsholm HTX	56.15	8.99	6
Himmerlev Gymnasium	55.66	12.11	18
Horsens Gymnasium	55.84	9.83	30
Køge Gymnasium	55.46	12.17	14
Langkær Gymnasium	56.19	10.11	-
Mariagerfjord Gymnasium	56.63	9.81	17
Marselisborg Gymnasium	56.14	10.2	19
Mulernes Legatskole	55.41	10.43	19
Nakskov Gymnasium og HF	54.84	11.14	12
Nørresundby Gymnasium og HF	57.07	9.94	22
Nykøbing Katedralskole	54.77	11.88	41
Ribe Katedralskole	55.33	8.76	25
Risskov Gymnasium	56.19	10.21	15
Rødkilde Gymnasium	55.71	9.55	5
Roskilde Gymnasium	55.64	12.08	26
Silkeborg Gymnasium	56.18	9.6	37
Skive Gymnasium	56.55	9.03	19
Sorø Akademi	55.43	11.56	39
Tørring Gymnasium	55.86	9.48	15
Varde Gymnasium	55.63	8.5	19
Vejles Tekniske Gymnasium	55.71	9.54	12
Vestfyns Gymnasium	55.27	10.11	26
Viborg Gymnasium og HF	56.46	9.45	10
Viby Gymnasium	56.11	10.15	7
Viby Teknisk Gymnasium	56.18	10.19	17
Virum Gymnasium	55.79	12.48	11
Total N			662

The age of participants ranged from 15 to 20 (median = 18) and their female-to-male ratio was 2.16. Questionnaire completeness was particularly high with 566 out of 662 (85.5%) students delivering at least 80% complete questionnaires (Fig 4B). Interestingly, female students delivered more complete questionnaires than male students (Mann-Whitney U = 52881, p = 0.01; Fig 4C), but no significant clustering was observed by age or parental education background (Kruskal-Wallis H test, p > 0.05; Fig 4D and 4E). Genetic and phenotypic data have been deposited at the European Genome-Phenome Archive (EGA; https://ega-archive.org) under accession number EGAS00001001868.

Modern Danish society accommodates different ethnic and cultural groups and this was also reflected in our sample, where ~4% of the participants were born in countries like Afghanistan, China, Ethiopia, Finland, Germany, Greenland, Iraq, Jordan, Korea, Kosovo, the Netherlands and Zambia. This number went up when we looked at grandparental origin, where 14.6% of the participants had at least one grandparent born outside Denmark. Four hundred seven (407) participants had their four grandparents born in Denmark (a typical inclusion criterion when analyzing historical patterns of genetic variation), whereas 131 participants had their four grandparents born in more specific regions (Table 2).

**Table 2:** Number of participants representing six well-defined geographic regions in Denmark.

Region	N (all four of the grandparents born in the same region)	N (at least three of the grandparents born in the same region)
Capital Region	4	14
Zealand	21	36
Funen	6	13
South Jutland	24	37
Central Jutland	48	89
North Jutland	28	48
Total	131	237

## Discussion

In many aspects, high schools are an ideal platform for promoting scientific literacy [9]. Experience has shown that high school students combine all the useful characteristics needed for successful scientific training: a genuine interest in novel technologies; the maturity to reflect upon ethical, legal, and social aspects of genetics; and the intellectual capacity and availability for such undertakings [10]. Partnerships with high schools, however, must be fast paced so that most participants can receive feedback on their involvement while they are still students. In this spirit, *Where Are You From*? was coordinated and delivered in an astonishingly short time (Fig 2), making a clear point that good scientific outreach does not need to be lengthy or prohibitively expensive.

Perhaps the best indicator of the success of our project was the warm response from students and their teachers, and the extensive publicity it drew in the media with coverage in nation-wide primetime television news channels. Indeed, approximately 600 students and their teachers–some traveling as far as 300 km–signed up for the second symposium, in which we presented preliminary results on genetic ancestry and related demographic topics. Even though this number is smaller compared to the first symposium (~780 registered attendees), it still reflects a high level of commitment to the project’s activities. During the second symposium, comprehension of the genomic concepts presented was assessed *in situ* by use of simple “fun questions”, which the students answered by use of clickers. A diploma with information about Y-DNA and/or mtDNA haplogroups and the percentage of Neanderthal ancestry was awarded to each participant. In addition, filmed and edited material from the two symposia was almost immediately released online (http://hvorkommerdufra.dk/materiale/) for use by schools across Denmark.

Despite the disengagement of a few partners in the course of the project (Fig 1), we managed to sample approximately one in 9,350 inhabitants of Denmark. To put this in context, a similar project in the UK (*People of the British Isles*) sampled one in 16,000 inhabitants [11]. With the help of the genetic data collected, we have studied the fine-scale population structure and historical genetic influences in Denmark [7].

The merit of our project lies in that (i) it enhances genomic literacy in high school students, one of the most dynamic and malleable demographics of the population and (ii) it points out that students, with the guidance of their teachers, can be as good a source of genetic data as other segments of the general population. Our high school students showed a commitment that is comparable to that of adult participants in medical studies [12]. As already mentioned, we took great care in securing voluntary and fully informed student enrolment. Yet, because the activity was carried out in a school context, we cannot discard sporadic cases of student participation due to real or, more probably, perceived pressure by peers and/or teachers.

Finally, we believe that future scientific outreach projects with a focus similar to that of *Where Are You From*? can benefit largely from incorporating more types of environmental and disease-related data. As showcased by the Precision Medicine Initiative, launched in early 2015 in the USA, such an endeavor is nowadays possible thanks to the increased user connectivity through social media and mobile devices that provide real-time measurements of variables such as glucose concentration, blood pressure and cardiac rhythm [4]. Aided by the technological and social advancements, and contingent on informed consent, future outreach projects and online initiatives, such as our very own *TheHonestGene*.*org*, can therefore be the ideal platform for recruiting participants for large-scale genomic studies.

## Conclusions

In sum, the high response rate of high school students to an online questionnaire in the context of a genetic outreach activity is a nontrivial finding, given that little is known about the engagement of adolescents in sample recruitment. Our endeavor sends out an encouraging message to geneticists who intend to design new population genetic projects, promoting, at the same time, genomic literacy among the youngest members of society.

## Supporting Information

S1 Table The online questionnaire answered by all participants in the *Where Are You From*? project. (DOCX)

S2 Table English translation of the participation statement signed by high school students or their parents/guardians. (DOCX)
