# Supplementary material for: Spitting for Science: Danish High School Students Commit to a Large-Scale Self-Reported Genetic Study
Source: PLoS One. 2016 Aug 29;11(8):e0161822. doi: 10.1371/journal.pone.0161822 (PMC5003382; doi:10.1371/journal.pone.0161822)
Supplement: S1 Table — (DOCX) [file pone.0161822.s001.docx]

**S1 Table.** The online questionnaire answered by all participants in the *Where Are You From?* project

| **Question** | **Answer type** |
| --- | --- |
| What is your name? | Free text |
| How old are you? | Free text |
| Which school are you from? | Dropdown menu |
| What is your kit number? (14 digits, no dashes or spaces) | Free text |
| Repeat kit number | Free text |
| Email address | Free text |
| Repeat email address | Free text |
| In which city were you born (country if outside Denmark)? | Free text |
| Which postal code (omit if outside Denmark)? | Free text |
| In which city was your father born (country if outside Denmark)? | Free text |
| Which postal code (omit if outside Denmark)? | Free text |
| In which city was your mother born (city, country if outside Denmark)? | Free text |
| Which postal code (omit if outside Denmark)? | Free text |
| In which city is your maternal grandmother born (city, country if outside Denmark)? | Free text |
| Which postal code (omit if outside Denmark)? | Free text |
| In which city is your maternal grandfather was born (city, country if outside Denmark)? | Free text |
| Which postal code (omit if outside Denmark)? | Free text |
| In which city is your paternal grandmother born (city, country if outside Denmark)? | Free text |
| Which postal code (omit if outside Denmark)? | Free text |
| In which city is your paternal grandfather was born (city, country if outside Denmark)? | Free text |
| Which postal code (omit if outside Denmark)? | Free text |
| Enter birthplace, land for as many as possible of your great–grandparents | Free text |
| Other knowledge of origin | Free text |
| Educational background – Mother | Dropdown menu |
| Educational background – Father | Dropdown menu |
| Educational background – Maternal grandmother | Dropdown menu |
| Educational background – Maternal grandfather | Dropdown menu |
| Educational background – Paternal grandmother | Dropdown menu |
| Educational background – Paternal grandfather | Dropdown menu |
| Which classmates are you most with at school? (Max. three) | Free text |
| Which classmates are you most with outside school? (Max. three) | Free text |
| What is your eye color? | Dropdown menu |
| What is your natural hair color? | Dropdown menu |
| What is your height in centimeters? | Free text |
| What is your weight in kg? | Free text |
