# Supplementary material for: Spitting for Science: Danish High School Students Commit to a Large-Scale Self-Reported Genetic Study
Source: PLoS One. 2016 Aug 29;11(8):e0161822. doi: 10.1371/journal.pone.0161822 (PMC5003382; doi:10.1371/journal.pone.0161822)
Supplement: S2 Table — (DOCX) [file pone.0161822.s002.docx]

**S2** **Table.** English translation of the participation statement signed by high school students or their parents/guardians

*Where are you from?* - Participation statement

*Where are you from?* is a joint teaching and research project at Aarhus University. Based on the genetic data of high school students from across the country, we will be able to write for the first time ever the genetic history of the Danish population. The project is unique in its character.

Data collection consists in that all participants provide a saliva sample that will be sent for analysis to a company in the United States. Each participant has to personally register their own genetic testing assay on the company’s website with their name, birthday and gender. The company will determine each participant’s DNA variation that is relevant to the project. Data will be transferred to the analysis group in Denmark, after which they will be permanently deleted from the company’s database. In Denmark, the data will be analyzed in order to determine the genetic history of all participants. No health-related data will be exchanged. The analysis group will process the data confidentially. All data will become anonymized before analysis results are released to high schools for educational use.

The project is led by Associate Professor Thomas Mailund, Professor Mikkel Heide Schierup and Professor Peter C. Kjærgaard, Aarhus University, together with Associate Professor Frank Grønlund Sørensen, Tørring Gymnasium. It is supported by the Ministry of Children and Youth. Further information is available at hvorkommerdufra.dk.

The undersigned hereby consent to participate in the project “Where are you from?”, including the supply of a saliva sample for DNA determination. With this signature, I declare acceptance of the above procedure and conditions.

Name and signature

Parent / guardian’s signature in case of participant age under 18 years

High school:

Teacher:

Class:
